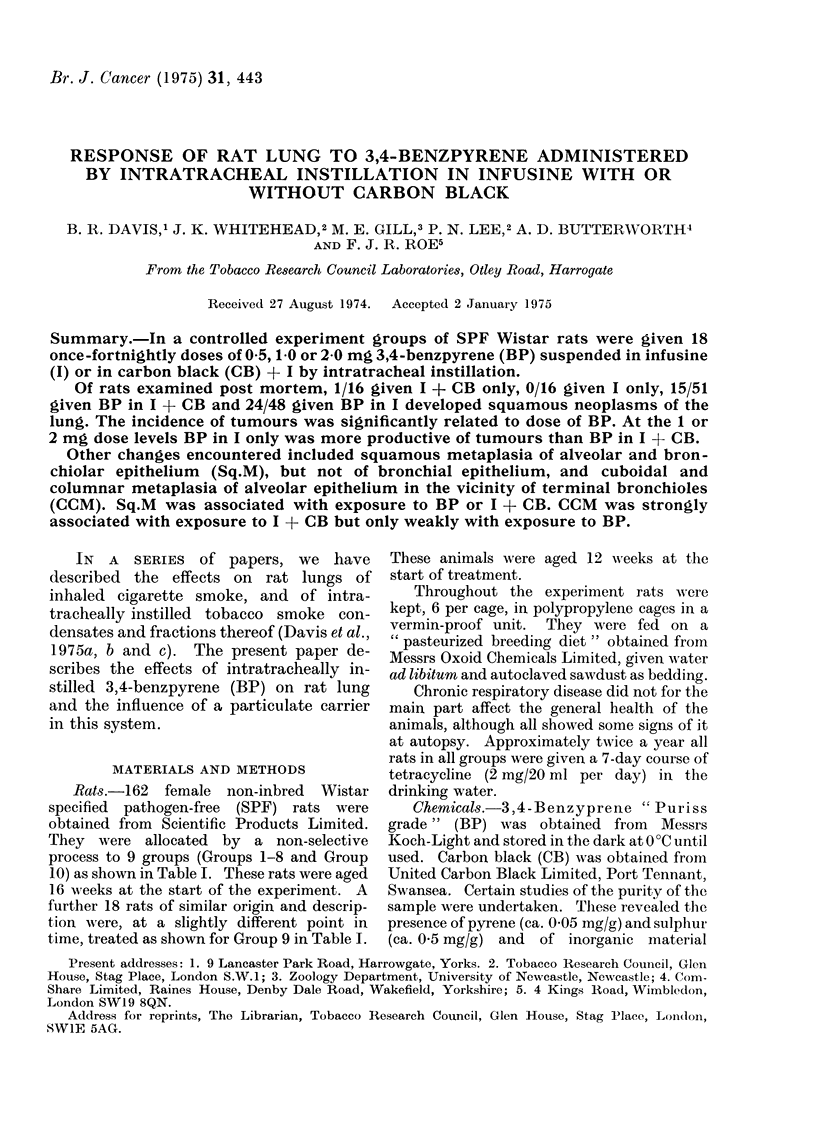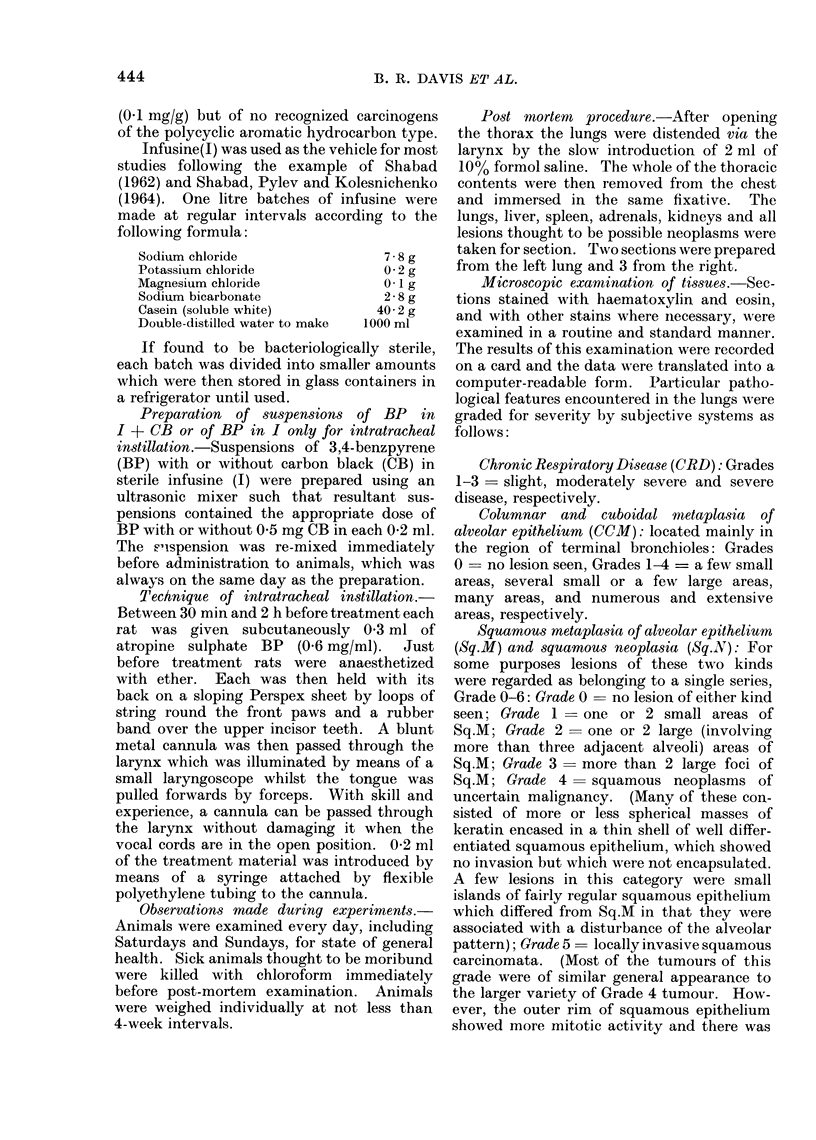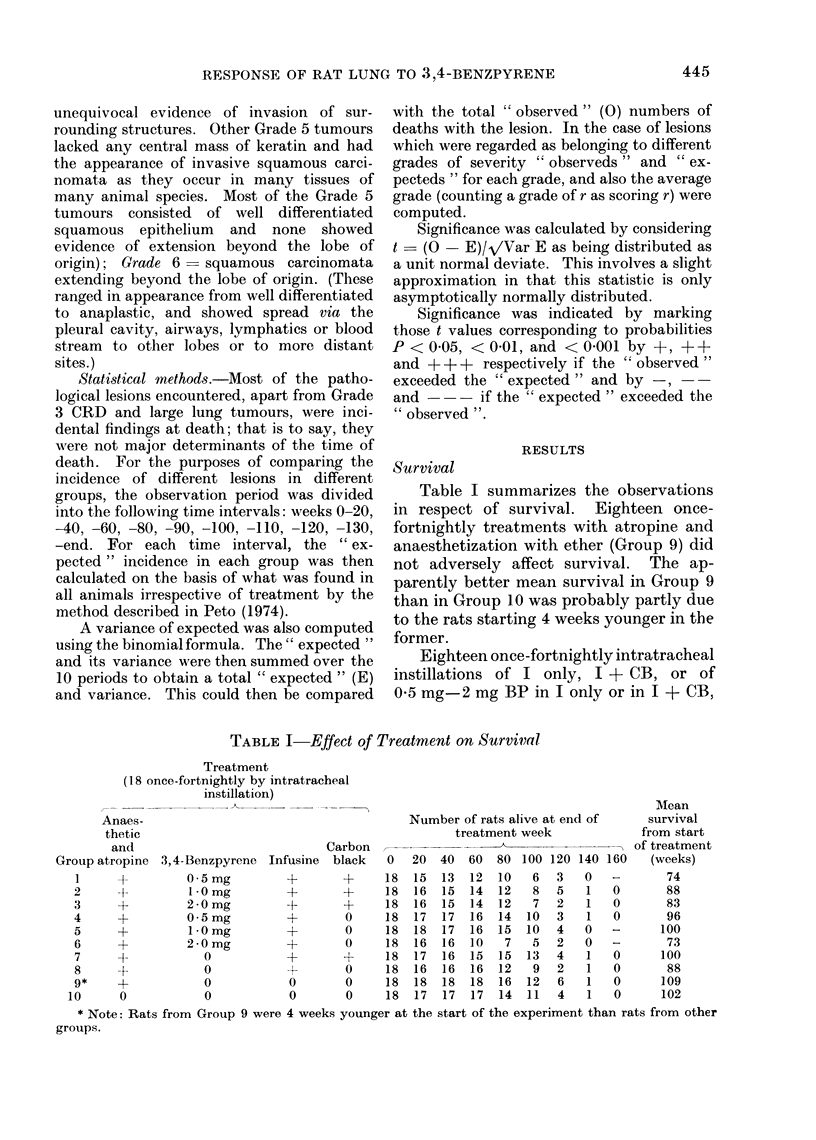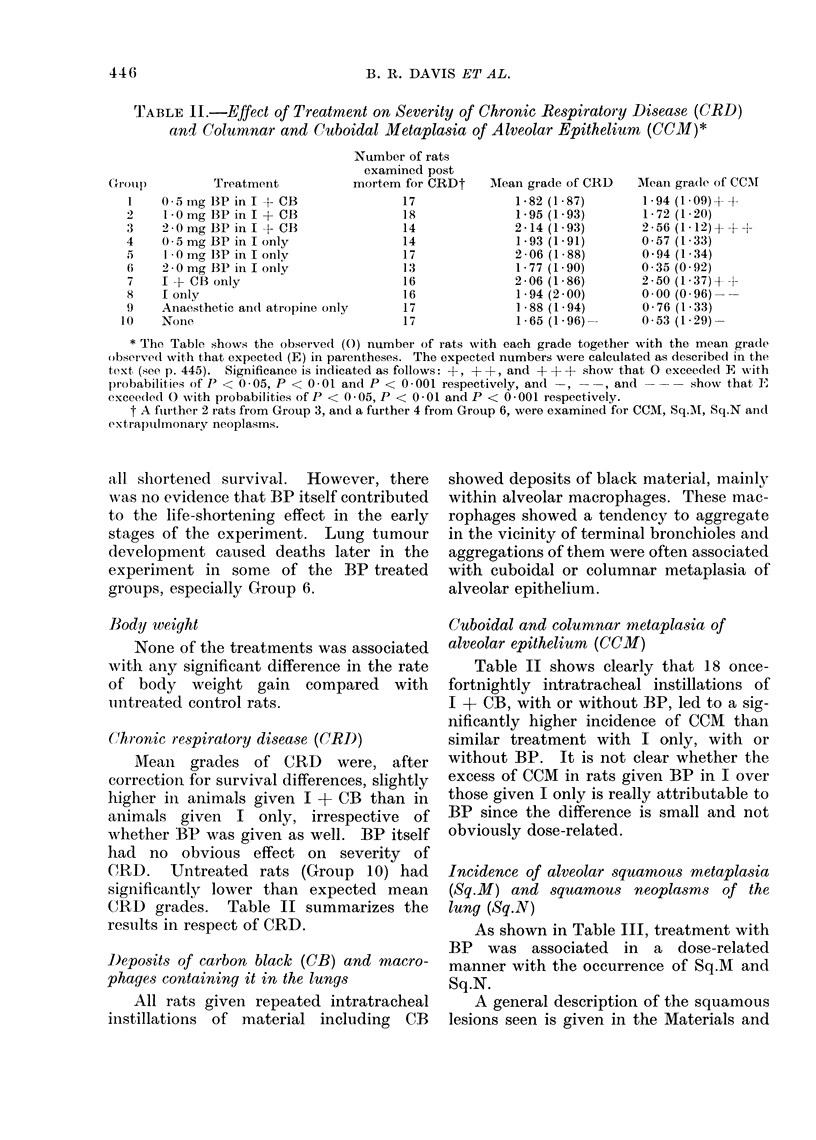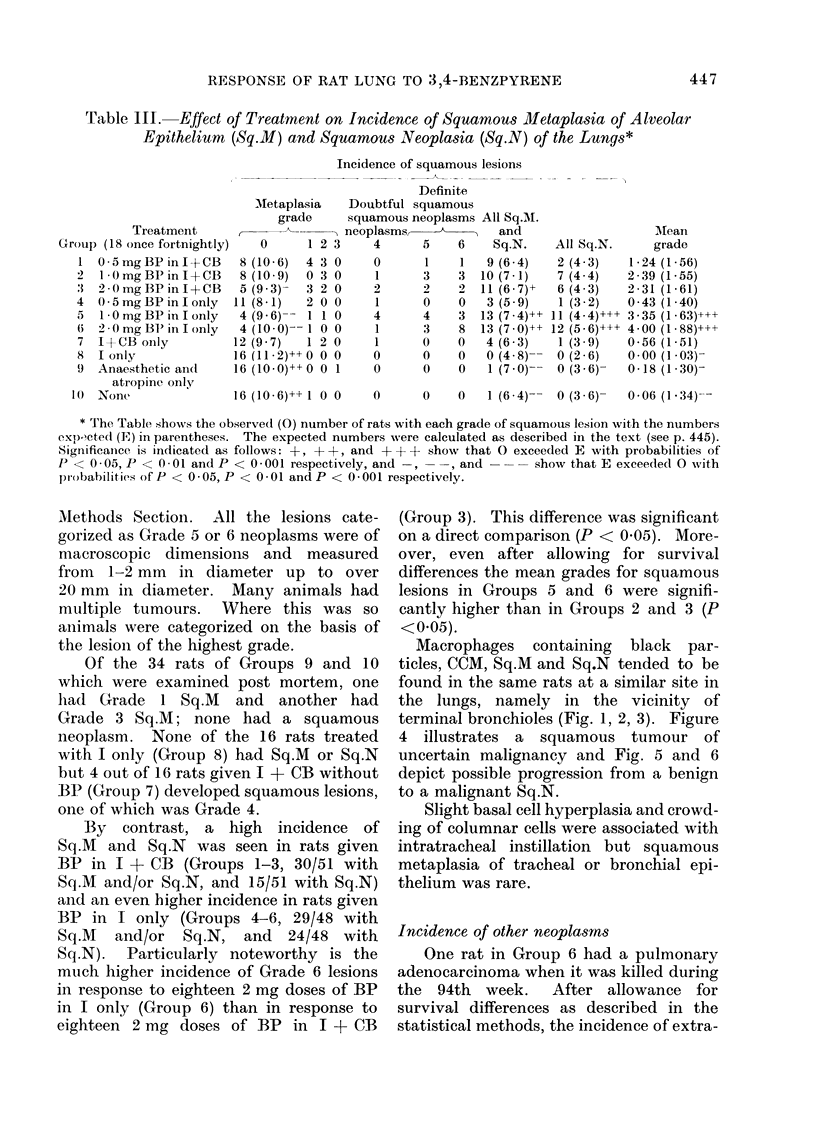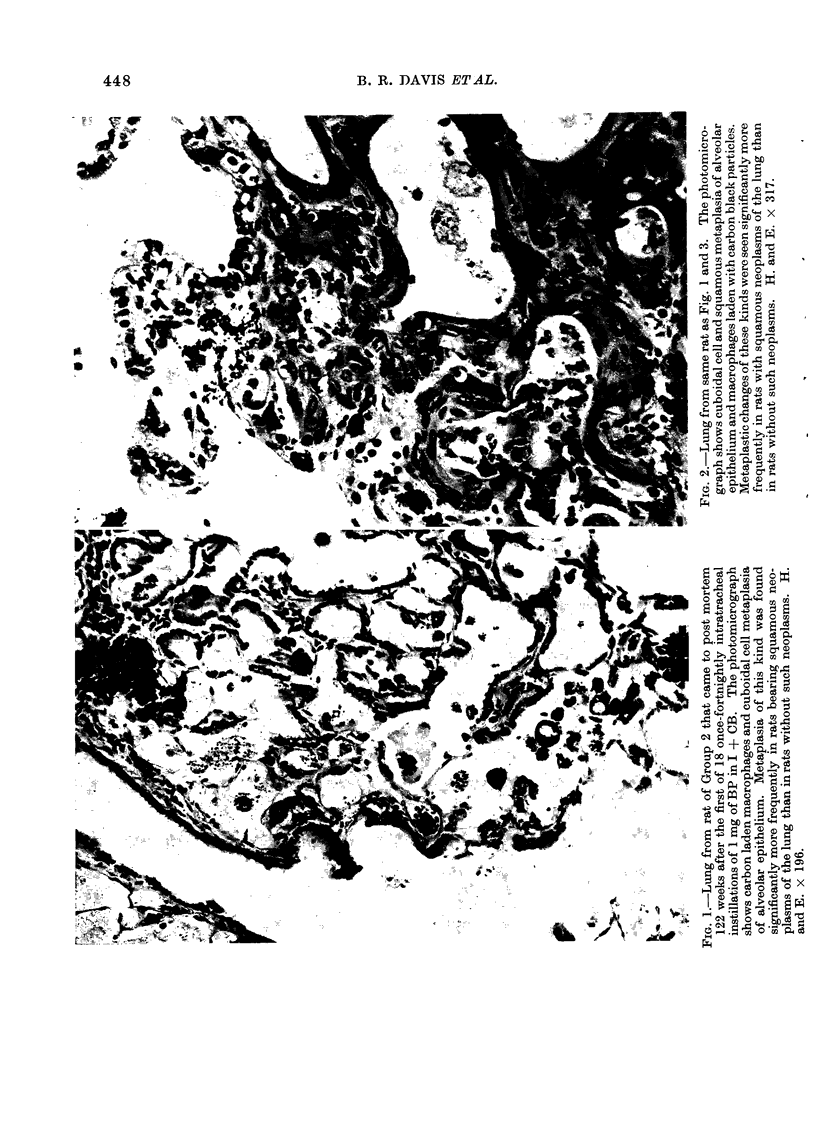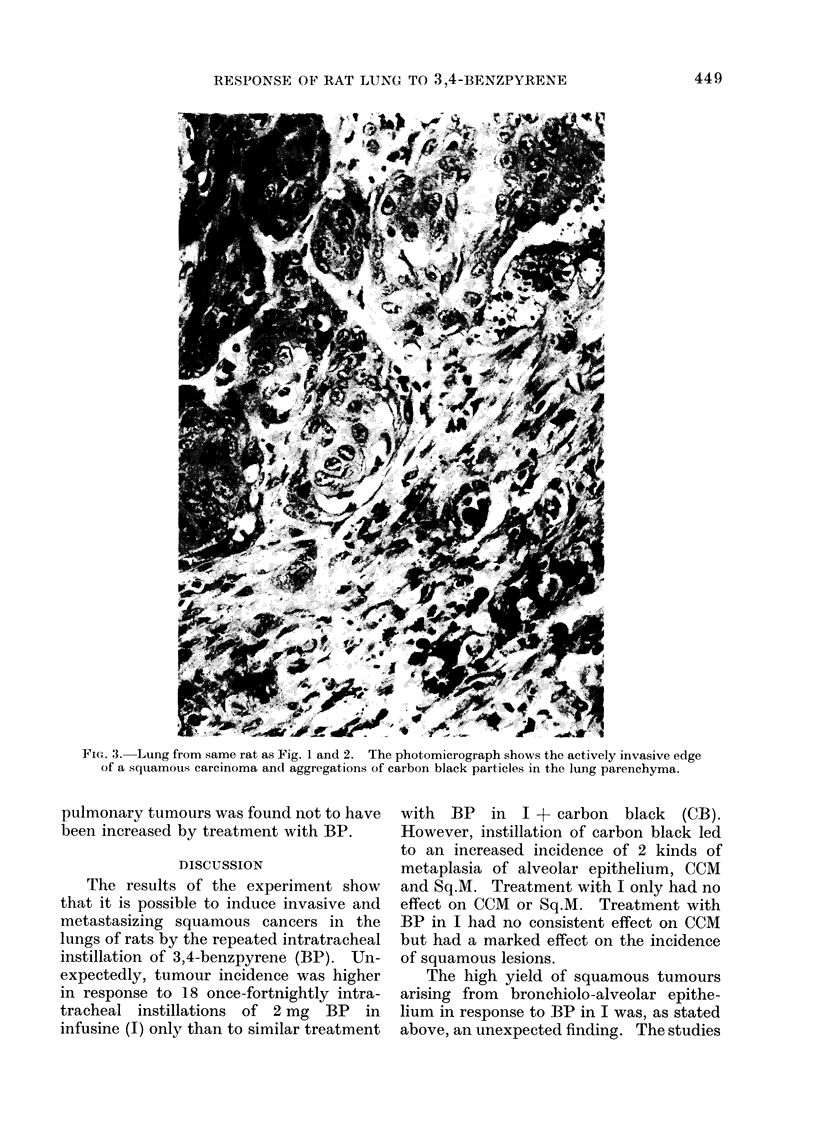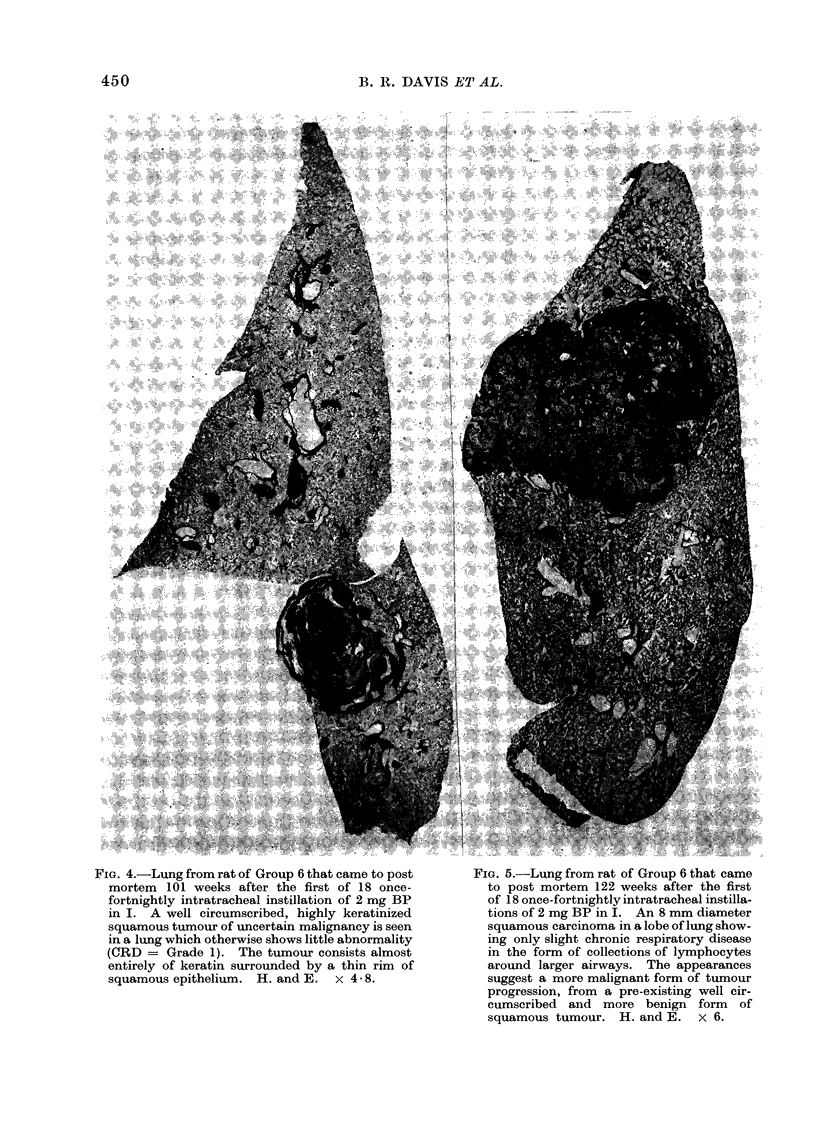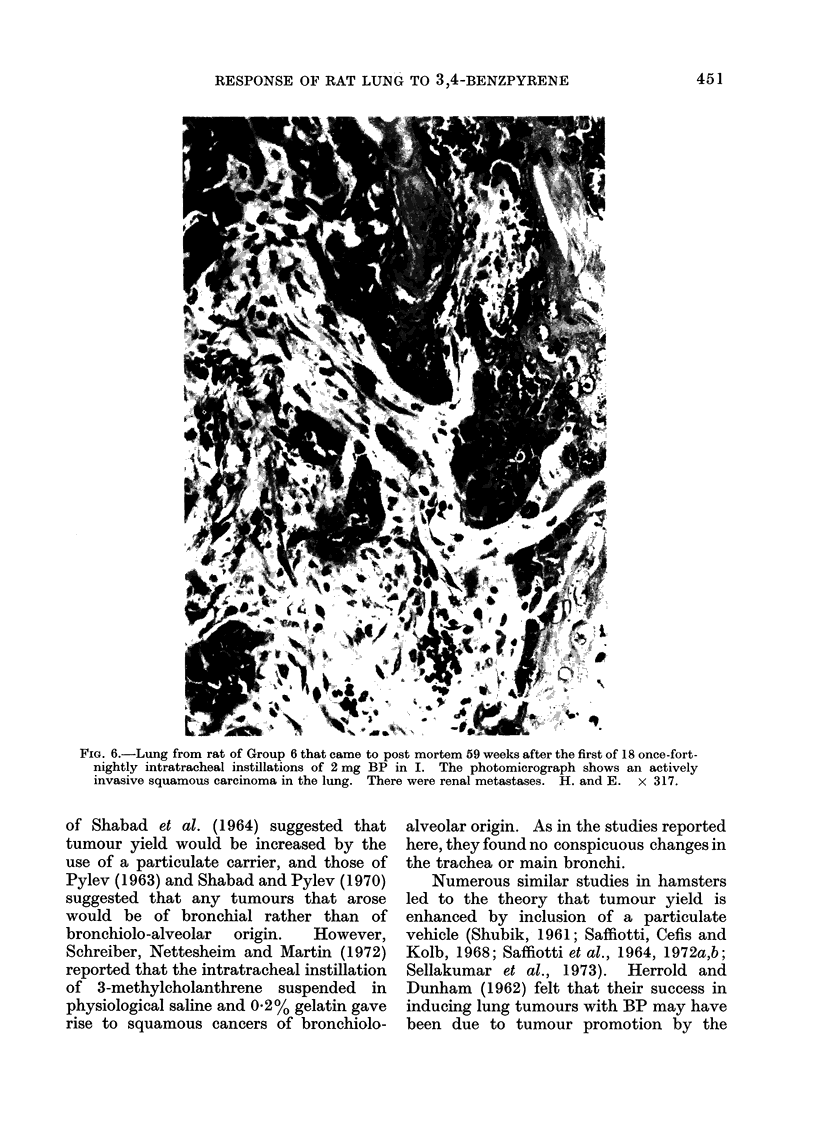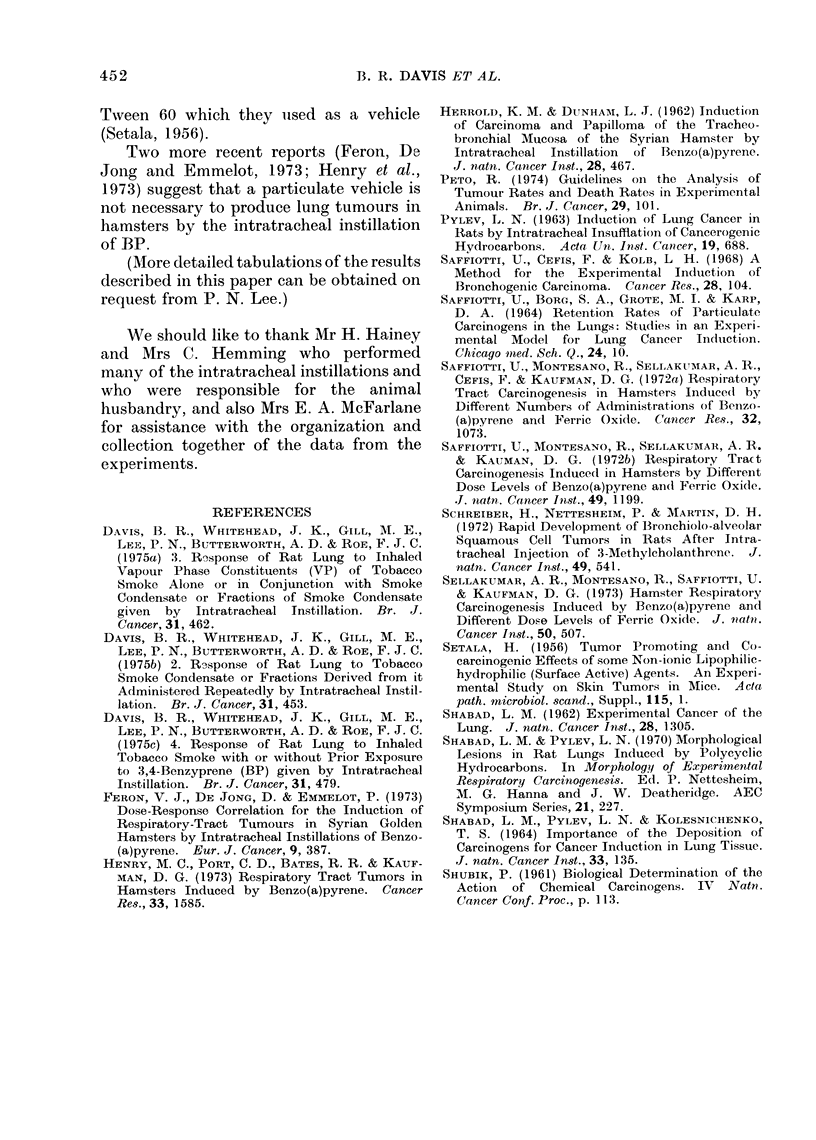# Response of rat lung to 3,4-benzpyrene administered by intratracheal instillation in infusine with or without carbon black.

**DOI:** 10.1038/bjc.1975.84

**Published:** 1975-04

**Authors:** B. R. Davis, J. K. Whitehead, M. E. Gill, P. N. Lee, A. D. Butterworth, F. J. Roe

## Abstract

**Images:**


*Br. J. Cancer* (1975) **31**, 443

### RESPONSE OF RAT LUNG TO 3,4-BENZPYRENE ADMINISTERED BY INTRATRACHEAL INSTILLATION IN INFUSINE WITH OR WITHOUT CARBON BLACK

B. R. DAVIS,[1] J. K. WHITEHEAD,[2] M. E. GILL,[3] P. N. LEE,[2] A. D. BUTTERWORTH[4] AND F. J. R. ROE[5]

*From the Tobacco Research Council Laboratories, Otley Road, Harrogate*

Received 27 August 1974. Accepted 2 January 1975

**Summary.—In a controlled experiment groups of SPF Wistar rats were given 18 once-fortnightly doses of 0·5, 1·0 or 2·0 mg 3,4-benzpyrene (BP) suspended in infusine (I) or in carbon black (CB) + I by intratracheal instillation.**

**Of rats examined post mortem, 1/16 given I + CB only, 0/16 given I only, 15/51 given BP in I + CB and 24/48 given BP in I developed squamous neoplasms of the lung. The incidence of tumours was significantly related to dose of BP. At the 1 or 2 mg dose levels BP in I only was more productive of tumours than BP in I + CB.**

**Other changes encountered included squamous metaplasia of alveolar and bronchiolar epithelium (Sq.M), but not of bronchial epithelium, and cuboidal and columnar metaplasia of alveolar epithelium in the vicinity of terminal bronchioles (CCM). Sq.M was associated with exposure to BP or I + CB. CCM was strongly associated with exposure to I + CB but only weakly with exposure to BP.**

In a series of papers, we have described the effects on rat lungs of inhaled cigarette smoke, and of intratracheally instilled tobacco smoke condensates and fractions thereof (Davis *et al.*, 1975*a*, *b* and *c*). The present paper describes the effects of intratracheally instilled 3,4-benzpyrene (BP) on rat lung and the influence of a particulate carrier in this system.

### MATERIALS AND METHODS

*Rats.*—162 female non-inbred Wistar specified pathogen-free (SPF) rats were obtained from Scientific Products Limited. They were allocated by a non-selective process to 9 groups (Groups 1–8 and Group 10) as shown in Table I. These rats were aged 16 weeks at the start of the experiment. A further 18 rats of similar origin and description were, at a slightly different point in time, treated as shown for Group 9 in Table I. These animals were aged 12 weeks at the start of treatment.

Throughout the experiment rats were kept, 6 per cage, in polypropylene cages in a vermin-proof unit. They were fed on a "pasteurized breeding diet" obtained from Messrs Oxoid Chemicals Limited, given water *ad libitum* and autoclaved sawdust as bedding.

Chronic respiratory disease did not for the main part affect the general health of the animals, although all showed some signs of it at autopsy. Approximately twice a year all rats in all groups were given a 7-day course of tetracycline (2 mg/20 ml per day) in the drinking water.

*Chemicals.*—3,4-Benzyprene "Puriss grade" (BP) was obtained from Messrs Koch-Light and stored in the dark at 0 °C until used. Carbon black (CB) was obtained from United Carbon Black Limited, Port Tennant, Swansea. Certain studies of the purity of the sample were undertaken. These revealed the presence of pyrene (ca. 0·05 mg/g) and sulphur (ca. 0·5 mg/g) and of inorganic material

Present addresses: 1. 9 Lancaster Park Road, Harrowgate, Yorks. 2. Tobacco Research Council, Glen House, Stag Place, London S.W.1; 3. Zoology Department, University of Newcastle, Newcastle; 4. Com-Share Limited, Raines House, Denby Dale Road, Wakefield, Yorkshire; 5. 4 Kings Road, Wimbledon, London SW19 8QN.

Address for reprints, The Librarian, Tobacco Research Council, Glen House, Stag Place, London, SW1E 5AG.

(0·1 mg/g) but of no recognized carcinogens of the polycyclic aromatic hydrocarbon type.

Infusine(I) was used as the vehicle for most studies following the example of Shabad (1962) and Shabad, Pylev and Kolesnichenko (1964). One litre batches of infusine were made at regular intervals according to the following formula:

| | |
|---|---|
| Sodium chloride | 7·8 g |
| Potassium chloride | 0·2 g |
| Magnesium chloride | 0·1 g |
| Sodium bicarbonate | 2·8 g |
| Casein (soluble white) | 40·2 g |
| Double-distilled water to make | 1000 ml |

If found to be bacteriologically sterile, each batch was divided into smaller amounts which were then stored in glass containers in a refrigerator until used.

*Preparation of suspensions of BP in I + CB or of BP in I only for intratracheal instillation.*—Suspensions of 3,4-benzpyrene (BP) with or without carbon black (CB) in sterile infusine (I) were prepared using an ultrasonic mixer such that resultant suspensions contained the appropriate dose of BP with or without 0·5 mg CB in each 0·2 ml. The suspension was re-mixed immediately before administration to animals, which was always on the same day as the preparation.

*Technique of intratracheal instillation.*—Between 30 min and 2 h before treatment each rat was given subcutaneously 0·3 ml of atropine sulphate BP (0·6 mg/ml). Just before treatment rats were anaesthetized with ether. Each was then held with its back on a sloping Perspex sheet by loops of string round the front paws and a rubber band over the upper incisor teeth. A blunt metal cannula was then passed through the larynx which was illuminated by means of a small laryngoscope whilst the tongue was pulled forwards by forceps. With skill and experience, a cannula can be passed through the larynx without damaging it when the vocal cords are in the open position. 0·2 ml of the treatment material was introduced by means of a syringe attached by flexible polyethylene tubing to the cannula.

*Observations made during experiments.*—Animals were examined every day, including Saturdays and Sundays, for state of general health. Sick animals thought to be moribund were killed with chloroform immediately before post-mortem examination. Animals were weighed individually at not less than 4-week intervals.

*Post mortem procedure.*—After opening the thorax the lungs were distended *via* the larynx by the slow introduction of 2 ml of 10% formol saline. The whole of the thoracic contents were then removed from the chest and immersed in the same fixative. The lungs, liver, spleen, adrenals, kidneys and all lesions thought to be possible neoplasms were taken for section. Two sections were prepared from the left lung and 3 from the right.

*Microscopic examination of tissues.*—Sections stained with haematoxylin and eosin, and with other stains where necessary, were examined in a routine and standard manner. The results of this examination were recorded on a card and the data were translated into a computer-readable form. Particular pathological features encountered in the lungs were graded for severity by subjective systems as follows:

*Chronic Respiratory Disease* (*CRD*)*:* Grades 1–3 = slight, moderately severe and severe disease, respectively.

*Columnar and cuboidal metaplasia of alveolar epithelium* (*CCM*)*:* located mainly in the region of terminal bronchioles: Grades 0 = no lesion seen, Grades 1–4 = a few small areas, several small or a few large areas, many areas, and numerous and extensive areas, respectively.

*Squamous metaplasia of alveolar epithelium* (*Sq.M*) *and squamous neoplasia* (*Sq.N*)*:* For some purposes lesions of these two kinds were regarded as belonging to a single series, Grade 0–6: *Grade* 0 = no lesion of either kind seen; *Grade* 1 = one or 2 small areas of Sq.M; *Grade* 2 = one or 2 large (involving more than three adjacent alveoli) areas of Sq.M; *Grade* 3 = more than 2 large foci of Sq.M; *Grade* 4 = squamous neoplasms of uncertain malignancy. (Many of these consisted of more or less spherical masses of keratin encased in a thin shell of well differentiated squamous epithelium, which showed no invasion but which were not encapsulated. A few lesions in this category were small islands of fairly regular squamous epithelium which differed from Sq.M in that they were associated with a disturbance of the alveolar pattern); *Grade* 5 = locally invasive squamous carcinomata. (Most of the tumours of this grade were of similar general appearance to the larger variety of Grade 4 tumour. However, the outer rim of squamous epithelium showed more mitotic activity and there was unequivocal evidence of invasion of surrounding structures. Other Grade 5 tumours lacked any central mass of keratin and had the appearance of invasive squamous carcinomata as they occur in many tissues of many animal species. Most of the Grade 5 tumours consisted of well differentiated squamous epithelium and none showed evidence of extension beyond the lobe of origin); *Grade* 6 = squamous carcinomata extending beyond the lobe of origin. (These ranged in appearance from well differentiated to anaplastic, and showed spread *via* the pleural cavity, airways, lymphatics or blood stream to other lobes or to more distant sites.)

*Statistical methods.*—Most of the pathological lesions encountered, apart from Grade 3 CRD and large lung tumours, were incidental findings at death; that is to say, they were not major determinants of the time of death. For the purposes of comparing the incidence of different lesions in different groups, the observation period was divided into the following time intervals: weeks 0–20, -40, -60, -80, -90, -100, -110, -120, -130, -end. For each time interval, the " expected " incidence in each group was then calculated on the basis of what was found in all animals irrespective of treatment by the method described in Peto (1974).

A variance of expected was also computed using the binomial formula. The " expected " and its variance were then summed over the 10 periods to obtain a total " expected " (E) and variance. This could then be compared with the total " observed " (O) numbers of deaths with the lesion. In the case of lesions which were regarded as belonging to different grades of severity " observeds " and " expecteds " for each grade, and also the average grade (counting a grade of $r$ as scoring $r$) were computed.

Significance was calculated by considering $t = (O - E)/\sqrt{\text{Var E}}$ as being distributed as a unit normal deviate. This involves a slight approximation in that this statistic is only asymptotically normally distributed.

Significance was indicated by marking those $t$ values corresponding to probabilities $P < 0{\cdot}05$, $< 0{\cdot}01$, and $< 0{\cdot}001$ by +, ++ and +++ respectively if the " observed " exceeded the " expected " and by —, — — and — — — if the " expected " exceeded the " observed ".

### RESULTS

### *Survival*

Table I summarizes the observations in respect of survival. Eighteen once-fortnightly treatments with atropine and anaesthetization with ether (Group 9) did not adversely affect survival. The apparently better mean survival in Group 9 than in Group 10 was probably partly due to the rats starting 4 weeks younger in the former.

Eighteen once-fortnightly intratracheal instillations of I only, I + CB, or of 0·5 mg—2 mg BP in I only or in I + CB,

TABLE I—*Effect of Treatment on Survival*

| Group | Treatment (18 once-fortnightly by intratracheal instillation): Anaesthetic and atropine | 3,4-Benzpyrene | Infusine | Carbon black | Number of rats alive at end of treatment week: 0 | 20 | 40 | 60 | 80 | 100 | 120 | 140 | 160 | Mean survival from start of treatment (weeks) |
|---|---|---|---|---|---|---|---|---|---|---|---|---|---|---|
| 1 | + | 0·5 mg | + | + | 18 | 15 | 13 | 12 | 10 | 6 | 3 | 0 | – | 74 |
| 2 | + | 1·0 mg | + | + | 18 | 16 | 15 | 14 | 12 | 8 | 5 | 1 | 0 | 88 |
| 3 | + | 2·0 mg | + | + | 18 | 16 | 15 | 14 | 12 | 7 | 2 | 1 | 0 | 83 |
| 4 | + | 0·5 mg | + | 0 | 18 | 17 | 17 | 16 | 14 | 10 | 3 | 1 | 0 | 96 |
| 5 | + | 1·0 mg | + | 0 | 18 | 18 | 17 | 16 | 15 | 10 | 4 | 0 | – | 100 |
| 6 | + | 2·0 mg | + | 0 | 18 | 16 | 16 | 10 | 7 | 5 | 2 | 0 | – | 73 |
| 7 | + | 0 | + | + | 18 | 17 | 16 | 15 | 15 | 13 | 4 | 1 | 0 | 100 |
| 8 | + | 0 | + | 0 | 18 | 16 | 16 | 16 | 12 | 9 | 2 | 1 | 0 | 88 |
| 9* | + | 0 | 0 | 0 | 18 | 18 | 18 | 18 | 16 | 12 | 6 | 1 | 0 | 109 |
| 10 | 0 | 0 | 0 | 0 | 18 | 17 | 17 | 17 | 14 | 11 | 4 | 1 | 0 | 102 |

* Note: Rats from Group 9 were 4 weeks younger at the start of the experiment than rats from other groups.

TABLE II.—*Effect of Treatment on Severity of Chronic Respiratory Disease (CRD) and Columnar and Cuboidal Metaplasia of Alveolar Epithelium (CCM)**

| Group | Treatment | Number of rats examined post mortem for CRD† | Mean grade of CRD | Mean grade of CCM |
|---|---|---|---|---|
| 1 | 0·5 mg BP in I + CB | 17 | 1·82 (1·87) | 1·94 (1·09)+ + |
| 2 | 1·0 mg BP in I + CB | 18 | 1·95 (1·93) | 1·72 (1·20) |
| 3 | 2·0 mg BP in I + CB | 14 | 2·14 (1·93) | 2·56 (1·12)+ + + |
| 4 | 0·5 mg BP in I only | 14 | 1·93 (1·91) | 0·57 (1·33) |
| 5 | 1·0 mg BP in I only | 17 | 2·06 (1·88) | 0·94 (1·34) |
| 6 | 2·0 mg BP in I only | 13 | 1·77 (1·90) | 0·35 (0·92) |
| 7 | I + CB only | 16 | 2·06 (1·86) | 2·50 (1·37)+ + |
| 8 | I only | 16 | 1·94 (2·00) | 0·00 (0·96)— — |
| 9 | Anaesthetic and atropine only | 17 | 1·88 (1·94) | 0·76 (1·33) |
| 10 | None | 17 | 1·65 (1·96)— | 0·53 (1·29)— |

* The Table shows the observed (O) number of rats with each grade together with the mean grade observed with that expected (E) in parentheses. The expected numbers were calculated as described in the text (see p. 445). Significance is indicated as follows: +, + +, and + + + show that O exceeded E with probabilities of $P < 0{\cdot}05$, $P < 0{\cdot}01$ and $P < 0{\cdot}001$ respectively, and —, — —, and — — — show that E exceeded O with probabilities of $P < 0{\cdot}05$, $P < 0{\cdot}01$ and $P < 0{\cdot}001$ respectively.

† A further 2 rats from Group 3, and a further 4 from Group 6, were examined for CCM, Sq.M, Sq.N and extrapulmonary neoplasms.

all shortened survival. However, there was no evidence that BP itself contributed to the life-shortening effect in the early stages of the experiment. Lung tumour development caused deaths later in the experiment in some of the BP treated groups, especially Group 6.

*Body weight*

None of the treatments was associated with any significant difference in the rate of body weight gain compared with untreated control rats.

*Chronic respiratory disease (CRD)*

Mean grades of CRD were, after correction for survival differences, slightly higher in animals given I + CB than in animals given I only, irrespective of whether BP was given as well. BP itself had no obvious effect on severity of CRD. Untreated rats (Group 10) had significantly lower than expected mean CRD grades. Table II summarizes the results in respect of CRD.

*Deposits of carbon black (CB) and macrophages containing it in the lungs*

All rats given repeated intratracheal instillations of material including CB showed deposits of black material, mainly within alveolar macrophages. These macrophages showed a tendency to aggregate in the vicinity of terminal bronchioles and aggregations of them were often associated with cuboidal or columnar metaplasia of alveolar epithelium.

*Cuboidal and columnar metaplasia of alveolar epithelium (CCM)*

Table II shows clearly that 18 once-fortnightly intratracheal instillations of I + CB, with or without BP, led to a significantly higher incidence of CCM than similar treatment with I only, with or without BP. It is not clear whether the excess of CCM in rats given BP in I over those given I only is really attributable to BP since the difference is small and not obviously dose-related.

*Incidence of alveolar squamous metaplasia (Sq.M) and squamous neoplasms of the lung (Sq.N)*

As shown in Table III, treatment with BP was associated in a dose-related manner with the occurrence of Sq.M and Sq.N.

A general description of the squamous lesions seen is given in the Materials and

Table III.—*Effect of Treatment on Incidence of Squamous Metaplasia of Alveolar Epithelium (Sq.M) and Squamous Neoplasia (Sq.N) of the Lungs**

| Group | Treatment (18 once fortnightly) | Incidence of squamous lesions: Metaplasia grade 0 | 1 | 2 | 3 | Doubtful squamous neoplasms 4 | Definite squamous neoplasms 5 | 6 | All Sq.M. and Sq.N. | All Sq.N. | Mean grade |
|---|---|---|---|---|---|---|---|---|---|---|---|
| 1 | 0·5 mg BP in I+CB | 8 (10·6) | 4 | 3 | 0 | 0 | 1 | 1 | 9 (6·4) | 2 (4·3) | 1·24 (1·56) |
| 2 | 1·0 mg BP in I+CB | 8 (10·9) | 0 | 3 | 0 | 1 | 3 | 3 | 10 (7·1) | 7 (4·4) | 2·39 (1·55) |
| 3 | 2·0 mg BP in I+CB | 5 (9·3)$^{-}$ | 3 | 2 | 0 | 2 | 2 | 2 | 11 (6·7)$^{+}$ | 6 (4·3) | 2·31 (1·61) |
| 4 | 0·5 mg BP in I only | 11 (8·1) | 2 | 0 | 0 | 1 | 0 | 0 | 3 (5·9) | 1 (3·2) | 0·43 (1·40) |
| 5 | 1·0 mg BP in I only | 4 (9·6)$^{--}$ | 1 | 1 | 0 | 4 | 4 | 3 | 13 (7·4)$^{++}$ | 11 (4·4)$^{+++}$ | 3·35 (1·63)$^{+++}$ |
| 6 | 2·0 mg BP in I only | 4 (10·0)$^{--}$ | 1 | 0 | 0 | 1 | 3 | 8 | 13 (7·0)$^{++}$ | 12 (5·6)$^{+++}$ | 4·00 (1·88)$^{+++}$ |
| 7 | I+CB only | 12 (9·7) | 1 | 2 | 0 | 1 | 0 | 0 | 4 (6·3) | 1 (3·9) | 0·56 (1·51) |
| 8 | I only | 16 (11·2)$^{++}$ | 0 | 0 | 0 | 0 | 0 | 0 | 0 (4·8)$^{--}$ | 0 (2·6) | 0·00 (1·03)$^{-}$ |
| 9 | Anaesthetic and atropine only | 16 (10·0)$^{++}$ | 0 | 0 | 1 | 0 | 0 | 0 | 1 (7·0)$^{--}$ | 0 (3·6)$^{-}$ | 0·18 (1·30)$^{-}$ |
| 10 | None | 16 (10·6)$^{++}$ | 1 | 0 | 0 | 0 | 0 | 0 | 1 (6·4)$^{--}$ | 0 (3·6)$^{-}$ | 0·06 (1·34)$^{--}$ |

* The Table shows the observed (O) number of rats with each grade of squamous lesion with the numbers expected (E) in parentheses. The expected numbers were calculated as described in the text (see p. 445). Significance is indicated as follows: +, + +, and + + + show that O exceeded E with probabilities of $P < 0{\cdot}05$, $P < 0{\cdot}01$ and $P < 0{\cdot}001$ respectively, and −, − −, and − − − show that E exceeded O with probabilities of $P < 0{\cdot}05$, $P < 0{\cdot}01$ and $P < 0{\cdot}001$ respectively.

Methods Section. All the lesions categorized as Grade 5 or 6 neoplasms were of macroscopic dimensions and measured from 1–2 mm in diameter up to over 20 mm in diameter. Many animals had multiple tumours. Where this was so animals were categorized on the basis of the lesion of the highest grade.

Of the 34 rats of Groups 9 and 10 which were examined post mortem, one had Grade 1 Sq.M and another had Grade 3 Sq.M; none had a squamous neoplasm. None of the 16 rats treated with I only (Group 8) had Sq.M or Sq.N but 4 out of 16 rats given I + CB without BP (Group 7) developed squamous lesions, one of which was Grade 4.

By contrast, a high incidence of Sq.M and Sq.N was seen in rats given BP in I + CB (Groups 1–3, 30/51 with Sq.M and/or Sq.N, and 15/51 with Sq.N) and an even higher incidence in rats given BP in I only (Groups 4–6, 29/48 with Sq.M and/or Sq.N, and 24/48 with Sq.N). Particularly noteworthy is the much higher incidence of Grade 6 lesions in response to eighteen 2 mg doses of BP in I only (Group 6) than in response to eighteen 2 mg doses of BP in I + CB (Group 3). This difference was significant on a direct comparison ($P < 0{\cdot}05$). Moreover, even after allowing for survival differences the mean grades for squamous lesions in Groups 5 and 6 were significantly higher than in Groups 2 and 3 ($P < 0{\cdot}05$).

Macrophages containing black particles, CCM, Sq.M and Sq.N tended to be found in the same rats at a similar site in the lungs, namely in the vicinity of terminal bronchioles (Fig. 1, 2, 3). Figure 4 illustrates a squamous tumour of uncertain malignancy and Fig. 5 and 6 depict possible progression from a benign to a malignant Sq.N.

Slight basal cell hyperplasia and crowding of columnar cells were associated with intratracheal instillation but squamous metaplasia of tracheal or bronchial epithelium was rare.

### *Incidence of other neoplasms*

One rat in Group 6 had a pulmonary adenocarcinoma when it was killed during the 94th week. After allowance for survival differences as described in the statistical methods, the incidence of extra-

FIG. 3.—Lung from same rat as Fig. 1 and 2. The photomicrograph shows the actively invasive edge of a squamous carcinoma and aggregations of carbon black particles in the lung parenchyma.

pulmonary tumours was found not to have been increased by treatment with BP.

### DISCUSSION

The results of the experiment show that it is possible to induce invasive and metastasizing squamous cancers in the lungs of rats by the repeated intratracheal instillation of 3,4-benzpyrene (BP). Unexpectedly, tumour incidence was higher in response to 18 once-fortnightly intratracheal instillations of 2 mg BP in infusine (I) only than to similar treatment with BP in I + carbon black (CB). However, instillation of carbon black led to an increased incidence of 2 kinds of metaplasia of alveolar epithelium, CCM and Sq.M. Treatment with I only had no effect on CCM or Sq.M. Treatment with BP in I had no consistent effect on CCM but had a marked effect on the incidence of squamous lesions.

The high yield of squamous tumours arising from bronchiolo-alveolar epithelium in response to BP in I was, as stated above, an unexpected finding. The studies

FIG. 4.—Lung from rat of Group 6 that came to post mortem 101 weeks after the first of 18 once-fortnightly intratracheal instillation of 2 mg BP in I. A well circumscribed, highly keratinized squamous tumour of uncertain malignancy is seen in a lung which otherwise shows little abnormality (CRD = Grade 1). The tumour consists almost entirely of keratin surrounded by a thin rim of squamous epithelium. H. and E. $\times$ 4·8.

FIG. 5.—Lung from rat of Group 6 that came to post mortem 122 weeks after the first of 18 once-fortnightly intratracheal instillations of 2 mg BP in I. An 8 mm diameter squamous carcinoma in a lobe of lung showing only slight chronic respiratory disease in the form of collections of lymphocytes around larger airways. The appearances suggest a more malignant form of tumour progression, from a pre-existing well circumscribed and more benign form of squamous tumour. H. and E. $\times$ 6.

FIG. 6.—Lung from rat of Group 6 that came to post mortem 59 weeks after the first of 18 once-fortnightly intratracheal instillations of 2 mg BP in I. The photomicrograph shows an actively invasive squamous carcinoma in the lung. There were renal metastases. H. and E. × 317.

of Shabad *et al.* (1964) suggested that tumour yield would be increased by the use of a particulate carrier, and those of Pylev (1963) and Shabad and Pylev (1970) suggested that any tumours that arose would be of bronchial rather than of bronchiolo-alveolar origin. However, Schreiber, Nettesheim and Martin (1972) reported that the intratracheal instillation of 3-methylcholanthrene suspended in physiological saline and 0·2% gelatin gave rise to squamous cancers of bronchiolo-alveolar origin. As in the studies reported here, they found no conspicuous changes in the trachea or main bronchi.

Numerous similar studies in hamsters led to the theory that tumour yield is enhanced by inclusion of a particulate vehicle (Shubik, 1961; Saffiotti, Cefis and Kolb, 1968; Saffiotti *et al.*, 1964, 1972*a*,*b*; Sellakumar *et al.*, 1973). Herrold and Dunham (1962) felt that their success in inducing lung tumours with BP may have been due to tumour promotion by the

Tween 60 which they used as a vehicle (Setala, 1956).

Two more recent reports (Feron, De Jong and Emmelot, 1973; Henry *et al.*, 1973) suggest that a particulate vehicle is not necessary to produce lung tumours in hamsters by the intratracheal instillation of BP.

(More detailed tabulations of the results described in this paper can be obtained on request from P. N. Lee.)

We should like to thank Mr H. Hainey and Mrs C. Hemming who performed many of the intratracheal instillations and who were responsible for the animal husbandry, and also Mrs E. A. McFarlane for assistance with the organization and collection together of the data from the experiments.

### REFERENCES

Davis, B. R., Whitehead, J. K., Gill, M. E., Lee, P. N., Butterworth, A. D. & Roe, F. J. C. (1975*a*) 3. Response of Rat Lung to Inhaled Vapour Phase Constituents (VP) of Tobacco Smoke Alone or in Conjunction with Smoke Condensate or Fractions of Smoke Condensate given by Intratracheal Instillation. *Br. J. Cancer*, **31**, 462.

Davis, B. R., Whitehead, J. K., Gill, M. E., Lee, P. N., Butterworth, A. D. & Roe, F. J. C. (1975*b*) 2. Response of Rat Lung to Tobacco Smoke Condensate or Fractions Derived from it Administered Repeatedly by Intratracheal Instillation. *Br. J. Cancer*, **31**, 453.

Davis, B. R., Whitehead, J. K., Gill, M. E., Lee, P. N., Butterworth, A. D. & Roe, F. J. C. (1975*c*) 4. Response of Rat Lung to Inhaled Tobacco Smoke with or without Prior Exposure to 3,4-Benzyprene (BP) given by Intratracheal Instillation. *Br. J. Cancer*, **31**, 479.

Feron, V. J., De Jong, D. & Emmelot, P. (1973) Dose-Response Correlation for the Induction of Respiratory-Tract Tumours in Syrian Golden Hamsters by Intratracheal Instillations of Benzo-(a)pyrene. *Eur. J. Cancer*, **9**, 387.

Henry, M. C., Port, C. D., Bates, R. R. & Kaufman, D. G. (1973) Respiratory Tract Tumors in Hamsters Induced by Benzo(a)pyrene. *Cancer Res.*, **33**, 1585.

Herrold, K. M. & Dunham, L. J. (1962) Induction of Carcinoma and Papilloma of the Tracheobronchial Mucosa of the Syrian Hamster by Intratracheal Instillation of Benzo(a)pyrene. *J. natn. Cancer Inst.*, **28**, 467.

Peto, R. (1974) Guidelines on the Analysis of Tumour Rates and Death Rates in Experimental Animals. *Br. J. Cancer*, **29**, 101.

Pylev, L. N. (1963) Induction of Lung Cancer in Rats by Intratracheal Insufflation of Cancerogenic Hydrocarbons. *Acta Un. Inst. Cancer*, **19**, 688.

Saffiotti, U., Cefis, F. & Kolb, L H. (1968) A Method for the Experimental Induction of Bronchogenic Carcinoma. *Cancer Res.*, **28**, 104.

Saffiotti, U., Borg, S. A., Grote, M. I. & Karp, D. A. (1964) Retention Rates of Particulate Carcinogens in the Lungs: Studies in an Experimental Model for Lung Cancer Induction. *Chicago med. Sch. Q.*, **24**, 10.

Saffiotti, U., Montesano, R., Sellakumar, A. R., Cefis, F. & Kaufman, D. G. (1972*a*) Respiratory Tract Carcinogenesis in Hamsters Induced by Different Numbers of Administrations of Benzo-(a)pyrene and Ferric Oxide. *Cancer Res.*, **32**, 1073.

Saffiotti, U., Montesano, R., Sellakumar, A. R. & Kauman, D. G. (1972*b*) Respiratory Tract Carcinogenesis Induced in Hamsters by Different Dose Levels of Benzo(a)pyrene and Ferric Oxide. *J. natn. Cancer Inst.*, **49**, 1199.

Schreiber, H., Nettesheim, P. & Martin, D. H. (1972) Rapid Development of Bronchiolo-alveolar Squamous Cell Tumors in Rats After Intratracheal Injection of 3-Methylcholanthrene. *J. natn. Cancer Inst.*, **49**, 541.

Sellakumar, A. R., Montesano, R., Saffiotti, U. & Kaufman, D. G. (1973) Hamster Respiratory Carcinogenesis Induced by Benzo(a)pyrene and Different Dose Levels of Ferric Oxide. *J. natn. Cancer Inst.*, **50**, 507.

Setala, H. (1956) Tumor Promoting and Co-carcinogenic Effects of some Non-ionic Lipophilic-hydrophilic (Surface Active) Agents. An Experimental Study on Skin Tumors in Mice. *Acta path. microbiol. scand.*, Suppl., **115**, 1.

Shabad, L. M. (1962) Experimental Cancer of the Lung. *J. natn. Cancer Inst.*, **28**, 1305.

Shabad, L. M. & Pylev, L. N. (1970) Morphological Lesions in Rat Lungs Induced by Polycyclic Hydrocarbons. In *Morphology of Experimental Respiratory Carcinogenesis.* Ed. P. Nettesheim, M. G. Hanna and J. W. Deatheridge. AEC Symposium Series, **21**, 227.

Shabad, L. M., Pylev, L. N. & Kolesnichenko, T. S. (1964) Importance of the Deposition of Carcinogens for Cancer Induction in Lung Tissue. *J. natn. Cancer Inst.*, **33**, 135.

Shubik, P. (1961) Biological Determination of the Action of Chemical Carcinogens. IV *Natn. Cancer Conf. Proc.*, p. 113.